# Renal Cell Carcinoma in Pregnancy: A Case Report of a Rare Diagnosis

**DOI:** 10.7759/cureus.76960

**Published:** 2025-01-05

**Authors:** Beatriz Ferreira, Ana Rita Alves, Ana Cláudia Rodrigues

**Affiliations:** 1 Obstetrics and Gynecology, Unidade Local de Saúde da Região de Leiria, Leiria, PRT

**Keywords:** cancer, diagnosis, pregnancy, renal cell carcinoma, urinary symptoms

## Abstract

Renal cell carcinoma is rarely reported in pregnant women. Diagnosis is often delayed because symptoms are similar to physiological changes and disorders of pregnancy. Each case should be discussed on an individual basis because there are no guidelines described for the clinical management of this disease during pregnancy. We report the case of a 37-year-old woman who presented to the emergency department at six weeks of gestation with left-sided flank pain and pollakiuria. Renal ultrasound revealed a 6 cm solid mass in the left kidney, suggestive of a renal neoplasm. Magnetic resonance imaging showed no signs of local or distant metastasis. The patient was treated with laparoscopic radical nephrectomy at 14 weeks of gestation without any complications. Histopathology confirmed the diagnosis of a renal clear-cell carcinoma. Progression of pregnancy was normal, and the patient gave birth to a healthy child at term. Follow-up examinations after four years demonstrated no cancer recurrence.

## Introduction

A diagnosis of cancer during pregnancy is a rare situation, with an estimated incidence of 0.1% [1-3]. The majority of cases reported are breast cancer, lymphoma, and cervical cancer [1,2]. Renal cell carcinoma is rarely seen in pregnant women, existing only in around 100 cases described in the literature [3,4]. It is a challenging diagnosis because patients can be completely asymptomatic or report symptoms similar to usual physiological changes or disorders of pregnancy [1,3,4]. Although surgical resection is the standard treatment for renal cell carcinoma, there are no guidelines for the clinical management of this disease in pregnant women [1,3]. Most cases described were treated with laparoscopic radical nephrectomy during pregnancy or immediately after birth [4,5]. The prognosis is generally good for both mother and child [1].

## Case presentation

**Figure 1:**
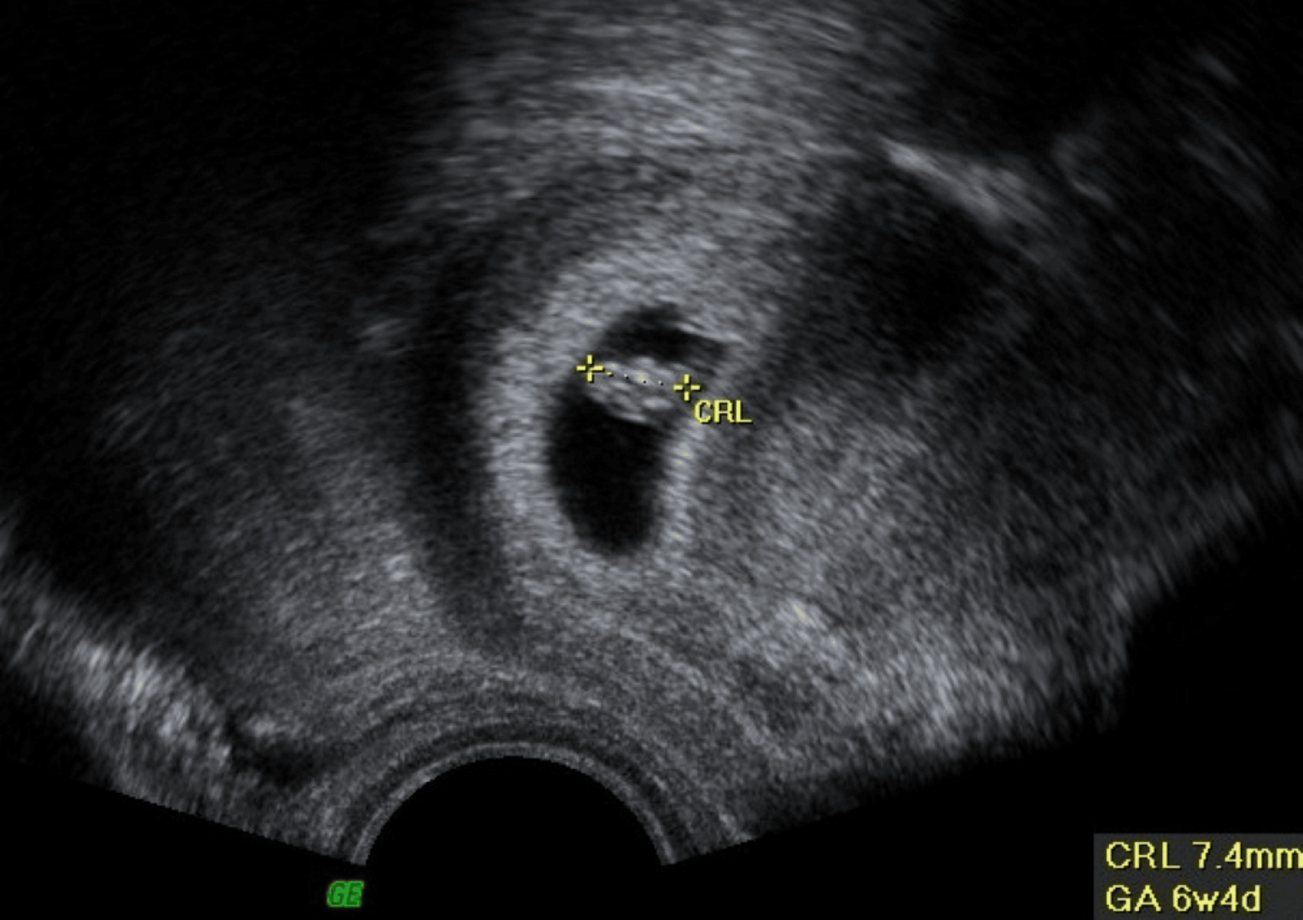
Transvaginal ultrasound

A 37-year-old female who had recently undergone infertility treatment presented to the emergency department with left-sided flank pain and pollakiuria for two days. She denied dysuria, suprapubic pain, or fever. Besides infertility, the patient had no relevant past medical history. Initial vital signs were a blood pressure of 111/65 mmHg, a heart rate of 80 bpm, and a body temperature of 37.6ºC. Physical examination revealed a soft, painless abdomen, normal vaginal discharge, and no palpable adnexal masses. Murphy's punch sign was positive on the left side. Transvaginal ultrasound revealed an intrauterine gestational sac with a live embryo with a crown-rump length compatible with six weeks of gestation (Figure 1).

Initial blood test results and urinalysis indicated moderate leukocytosis, elevated C-reactive protein, normal baseline creatinine level, and microscopic hematuria (Table 1). 

**Table 1:** Summary of the blood tests and urinalysis of the patient

	Normal range	Patient results
Hematology		
White blood cells	4.0 to 10.0 K/mm^3^	20.0 K/mm^3^
Hemoglobin	11.5 to 16.0 gm/dL	13.8 gm/dL
Platelets	150 to 500 K/mm^3^	334 K/mm^3^
Chemistry		
Urea	17 to 43 mg/dL	17 mg/dL
Creatinine	0.51 to 0.95 mg/dL	0.64 mg/dL
C-reactive protein	< 5.0 mg/L	43.9mg/L
Urinalysis		
Urine blood	negative	positive
Urine nitrate	negative	negative
Urine white blood cells	0 to 5/high power field	25/high power field

**Figure 2:**
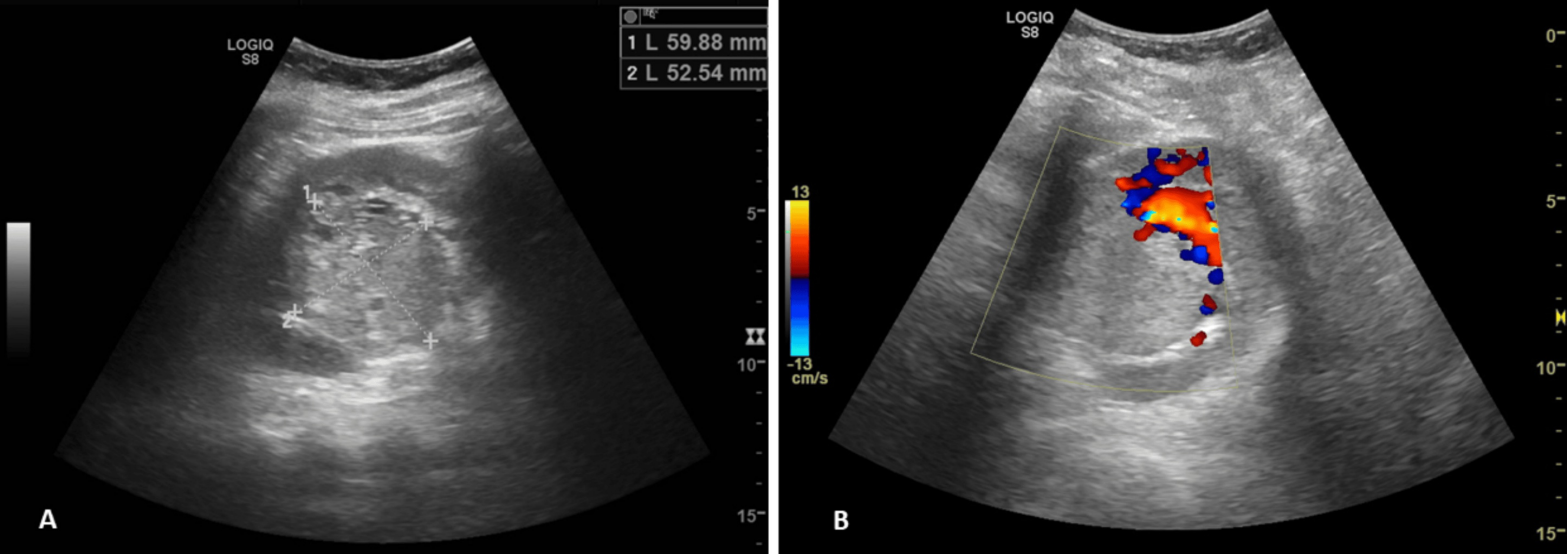
Ultrasound appearance of the renal mass (A) General appearance and dimensions of the renal mass; (B) Color Doppler revealing vascularization in the periphery of the lesion.

A renal ultrasound was performed and showed a 6 cm solid lesion in the upper pole of the left kidney. The appearance was highly suggestive of a renal neoplasm (Figure 2).

**Figure 3:**
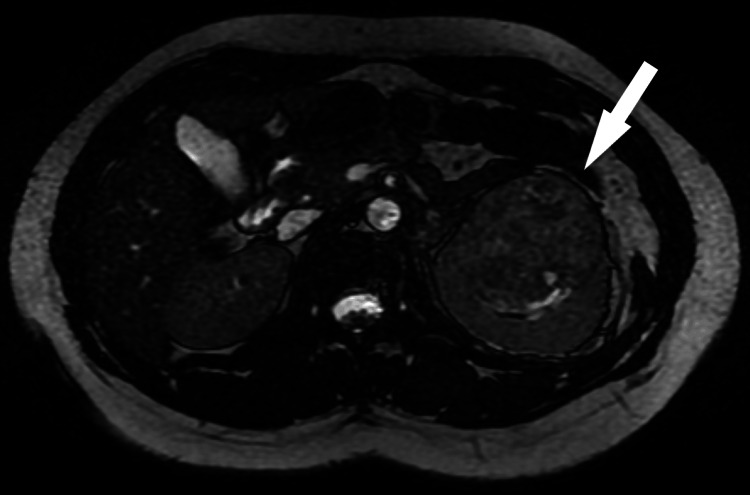
Magnetic resonance imaging of the abdomen showing the renal carcinoma (arrow)

A supplemental magnetic resonance imaging confirmed the presence of a 7.7x5.7x6 cm mass in the left kidney with no signs of lymphadenopathy or metastasis (Figure 3). 

**Figure 4:**
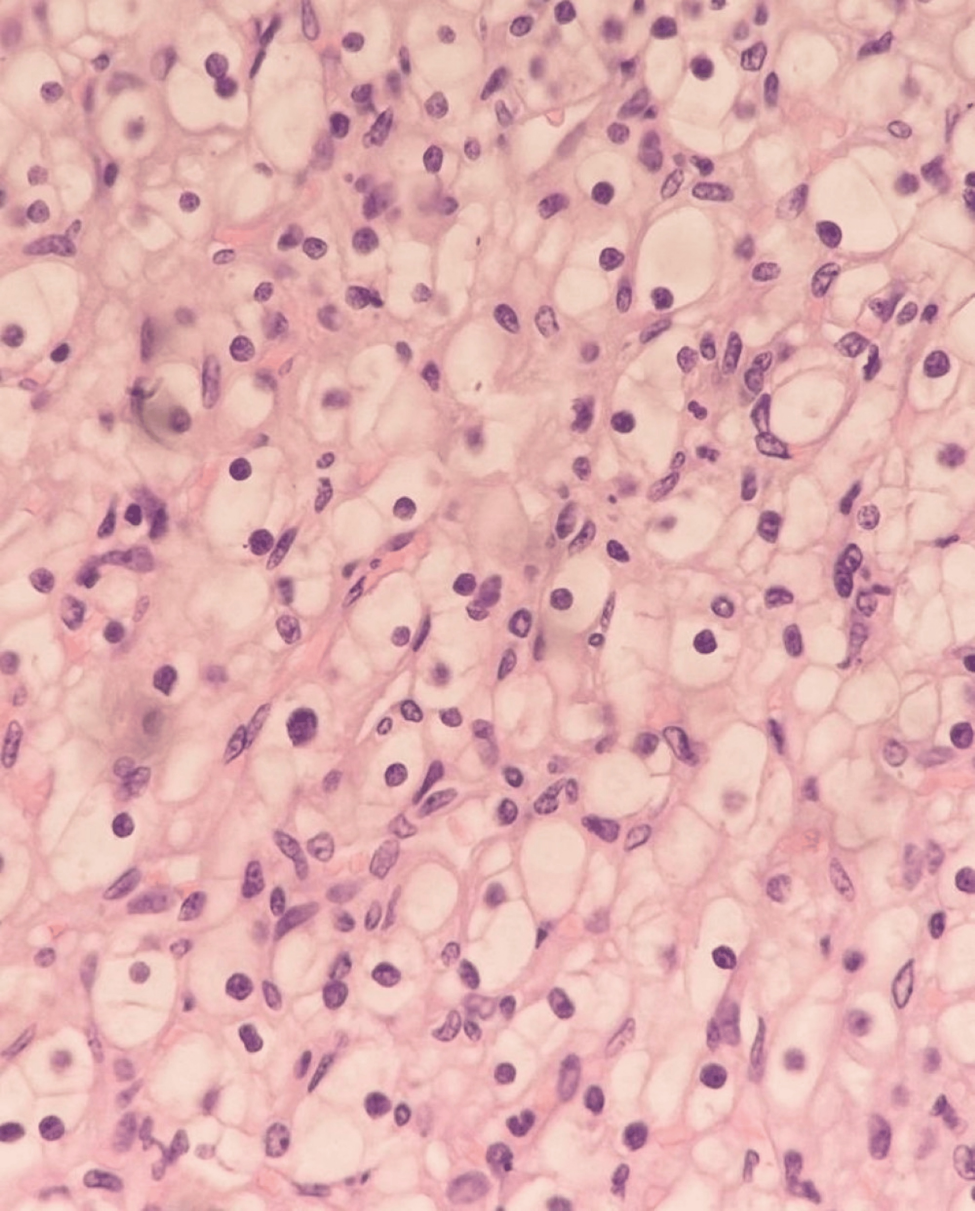
Histology showing the renal clear-cell carcinoma (400x magnification)

During the initial investigation, the patient was hospitalized, given a course of antibiotics, and discharged home within five days, clinically better. The case was discussed with the urology and anesthesiology departments, and the patient was scheduled for laparoscopic radical nephrectomy at 14 weeks of gestation. A transperitoneal approach was performed without any complications. The pathology report confirmed a renal clear-cell carcinoma with free resection margins, pathologic stage pT1b (Figure 4). 

Follow-up urology consultation revealed a favorable postoperative recovery, and the patient continued on a periodic obstetric control until delivery. A term labor induction was conducted because an obstetric routine at 37 weeks of pregnancy revealed a small-for-gestational-age fetus. Delivery was vaginal with no complications, and the child was developmentally normal.

**Figure 5:**
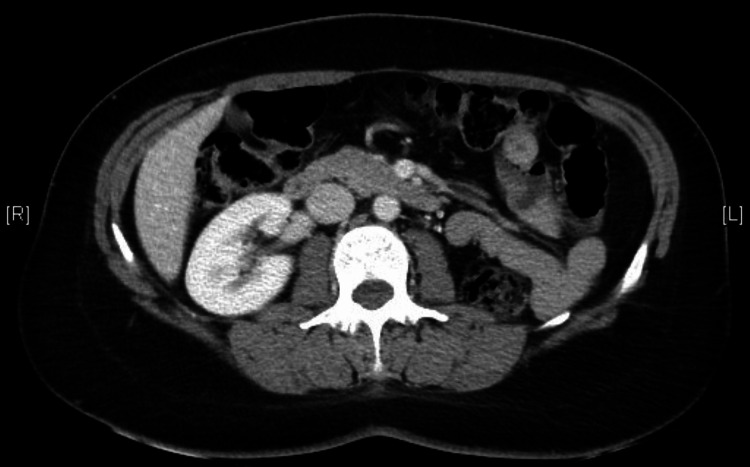
Follow-up computed tomography scan

The patient maintained urologic surveillance for four years, with follow-up computed tomography scans demonstrating no tumor recurrence (Figure 5). 

## Discussion

Renal cell carcinoma represents 3% of all malignancies, affecting mostly males in their sixth decade [1,5,6]. Modifiable risk factors include obesity, smoking, and hypertension [1,2]. Pregnancy itself is not considered a risk factor, but incidental findings during routine ultrasound examinations are responsible for an increase in the detection rates [2]. Nevertheless, gestational urologic cancer is extremely rare, affecting approximately 13 of 1,000,000 pregnancies [7,8]. Half of all urologic cancers detected during pregnancy are renal cell carcinomas, and the clear-cell type is the most frequent variant, accounting for 95% of all cases [6,9,10].

The diagnosis of gestational renal cell carcinoma is difficult because symptoms can be attributable to physiological changes and disorders of pregnancy, such as calculi, urinary tract infection, or preeclampsia [1,4]. Although the main complaints are flank pain, hematuria, and hypertension, most cases are completely asymptomatic, and the classical triad of symptoms (flank pain, palpable mass, and hematuria) is rarely seen [1,4,7]. Imaging technology is essential to confirm this diagnosis, but potential teratogenic effects need to be considered when pregnancy is suspected [2,4,7]. Ultrasound is widely used for obstetric evaluation, and it remains the safest complementary exam for acute abdominal conditions during pregnancy [1]. It is a good screening tool for renal cell carcinoma, achieving a high sensitivity for renal masses greater than 3 cm [2,4,7]. In the general population, computed tomography is the exam of choice to confirm the diagnosis of a renal neoplasm and the stage of the disease [1,3]. However, radiation exposure is high and can compromise fetal development in pregnant women [1]. A safe alternative for these patients is magnetic resonance imaging, as described in the cases already reported [3,4,7].

There are no guidelines for the treatment of renal cell carcinoma diagnosed during pregnancy [1,7]. A multidisciplinary team must evaluate each case considering tumor characteristics and obstetric aspects [1-3,7]. It is important to assess tumor size, stage of disease, patient's general health, timing of diagnosis, and fetus's chance of survival and well-being [2]. Gestational age at diagnosis is the most relevant obstetric factor to consider because it establishes the rate of survival for the fetus and influences the surgical technique when indicated [1,3]. Regarding tumor characteristics, it is known that every additional centimeter of tumor dimensions adds 25% more risk for distant metastasis [1,3]. Also, a persisting renal mass can compromise the well-being of both mother and fetus as it can complicate spontaneous bleeding, rupture, thrombosis, or ureteral compression [3]. That is why surgical resection is the standard treatment for renal cell carcinoma, and radical nephrectomy is the intervention of choice for most stages of the disease, even in pregnant women [1,4].

Studies suggest laparoscopy as the preferred approach to manage several acute abdominal disorders in pregnancy, with no relevant complications reported [2-4]. The most common procedures described are appendicectomy, cholecystectomy, and management of adnexal masses [6,8,10]. The benefits of laparoscopy are the same as those described for the general population, including reduced blood loss, less pain and need for analgesics, faster return to normal bowel function, and quicker postoperative recovery [2,4,5]. O’Connor and colleagues reported in 2004 that the first laparoscopic nephrectomy was performed in a pregnant woman [2,3,10]. Since then, it has been described in several cases of laparoscopic nephrectomy without any serious complications [3,9]. Laparoscopic nephrectomy can be performed by a transperitoneal or a retroperitoneal route, both feasible in pregnancy and selected by the preference of the surgeon [1-4]. The retroperitoneal route allows more direct access to the kidney, but the working space is narrower than in the transperitoneal route [1,2]. The ideal timing of surgical intervention lacks consensus, and several approaches have been described concerning the three trimesters of pregnancy [1-3,9]. The second trimester of pregnancy is the most cited period to perform the surgery because spontaneous miscarriage is higher in the first trimester and the risk of preterm labor increases with gestational age [1-5,10]. Pregnancy interruption may also be considered when diagnosis is made earlier or later in gestation [1,2]. Therefore, risks and benefits must be assessed for both mother and fetus in every trimester [1,3].

Prognosis is excellent for the general population as well as for pregnant women and their fetuses [1,3,4]. Prompt diagnosis and expedited multidisciplinary management are essential to predict prognosis, as radical nephrectomy is curative in the majority of cases [2,4].

In this clinical case, prompt diagnosis was essential for the patient to have a good prognosis. Although flank pain and microscopic hematuria were suggestive of calculi or acute pyelonephritis, the renal ultrasound revealed a solid mass, suggestive of a renal neoplasm. A complementary magnetic resonance imaging showed no more lesions or lymphatic involvement. Surgical resection by a laparoscopic approach was proposed to the patient to be executed at the beginning of the second trimester. That timing was defined after considering the slow tumor growth rate, low risk for metastatic spread, and safety of the fetus. Pregnancy could end on its own during the first trimester (considering the high risk of spontaneous abortions) or could be medically interrupted due to a major malformation of the fetus detected on the first-trimester ultrasound. These situations would prevent surgery from occurring during pregnancy and potentially complicate fetal organogenesis. Surgery was performed by a transperitoneal route as it allowed more space to work with. No major complications occurred in the postoperative period and progression of pregnancy. 

Flank pain in a pregnant patient, especially when associated with another urinary symptom or sign, should prompt a clinical investigation for renal pathology. It was fortunate that this patient referred flank pain because diagnosis is often delayed by the number of patients that are completely asymptomatic.

## Conclusions

The diagnosis of renal cell carcinoma in a pregnant woman is challenging because symptoms, when reported, are similar to physiological changes and disorders of pregnancy. Suspicion is higher if there is flank pain, hematuria, or a presenting palpable mass. Renal ultrasound is the exam of choice to first characterize a renal mass, followed by magnetic resonance imaging to stage the disease in the pregnant population. Regarding the rarity of this diagnosis in pregnancy and the absence of guidelines about the treatment, each case should be managed on an individual basis, guided by a multidisciplinary team.
